# Supplementary material for: Cardiovascular Risk in Patients with Dyslipidemia and Their Degree of Control as Perceived by Primary Care Physicians in a Survey—TERESA-Opinion Study
Source: Int J Environ Res Public Health. 2023 Jan 29;20(3):2388. doi: 10.3390/ijerph20032388 (PMC9915170; doi:10.3390/ijerph20032388)
Supplement: Supplementary file 1 [file ijerph-20-02388-s001.zip › ijerph-2140087-supplementary.pdf]

Questionnaire for Physicians

Details of Physicians

Age ___________ years

Autonomous community ___________ (list)

Population:

- ☐ >20,000 inhabitants
- ☐ 5,000-20,000 inhabitants
- ☐ <5,000 inhabitants

Type of healthcare centre:

- ☐ Health centre
- ☐ Rural practice

Teaching centre (YES/NO)

Attending physician (YES/NO)

Tutor of degree students (YES/NO)

Number of patients registered at your practice: ___________

- ☐ <500 patients
- ☐ 500-1000 patients
- ☐ 1000-1500 patients
- ☐ >1500 patients

Situation of dyslipidemia and its treatment

From the patients on your list, what percentage of patients older than 40 do you consider to have dyslipidemia?

__________%

Among these patients, in what percentage do you use the following therapeutic strategies?

High-intensity statin and ezetimibe	%
Moderate-intensity statin and ezetimibe	%
High-intensity statin	%
Moderate-intensity statin	%
Low-intensity statin	%
Fibrate, in monotherapy	%
Ezetimibe, in monotherapy	%
Bempedoic acid	%
Others (for instance, nutraceuticals, ...)	%

* High-intensity statins*: atorvastatin 80mg and rosuvastatin 20mg*
^ Moderate-intensity statins*: fluvastatin prolib (80mg), pravastatin (40mg), lovastatin (40mg), simvastatin (10mg, 20mg and 40mg), pitavastatin (1mg, 2mg and 4mg), atorvastatin (10mg, 20mg and 40mg) and rosuvastatin (5mg and 10mg) simvastatin*
# Low-intensity statins*: Fluvastatin (10mg and 20mg), pravastatin (10mg and 20mg) and lovastatin (20mg)*
Nutraceuticals: monacolin K, policosanols, soy sterols.

Indicate, if any, the number/percentage of patients in your list who are treated with PCSK9 inhibitors __________%

Of your patients with dyslipidemia older than 80, what percentage is being treated with statins

__________%

Indicate in which percentage the patients on your list with lipid-lowering treatment are distributed:

Recurring events in the last 2 years	%
Very high risk	%
High risk	%

Moderate risk	%
Low risk	%

Arrange in decreasing order, which comorbidities you consider most frequently associated with dyslipidemia:

Diabetes Mellitus	
Obesity	
Coronary heart disease	
Cerebrovascular disease	
Peripheral artery disease	
Hypertension	
Chronic kidney disease	

Indicate what percentage of your patients with dyslipidemia present the following comorbidities:

Diabetes Mellitus	%
Cardiovascular disease	%
Diabetes Mellitus and cardiovascular disease	%
Chronic kidney disease	%

Arrange in decreasing order, the statins you use most:

Rosuvastatin 40mg	
Rosuvastatin 30mg	
Rosuvastatin 20mg	
Rosuvastatin 10mg	
Rosuvastatin 5mg	
Atorvastatin 80mg	
Atorvastatin 60mg	

Atorvastatin 40mg	
Atorvastatin 30mg	
Atorvastatin 20mg	
Atorvastatin 10mg	
Pitavastatin 4mg	
Pitavastatin 2mg	
Pitavastatin 1mg	
Simvastatin 40mg	
Simvastatin 20mg	
Simvastatin 10 mg	
Lovastatin 40mg	
Lovastatin 20mg	
Pravastatin 40mg	
Pravastatin 20mg	
Pravastatin 10mg	
Fluvastatin prolib 80mg	
Fluvastatin 40mg	
Fluvastatin 20mg	
None	

What % of patients do you have with fixed combination treatment?

Simvastatin + Ezetimibe	%
Atorvastatin + Ezetimibe	%
Rosuvastatin + Ezetimibe	%
Statin + Fibrate	%
Bempedoic + Ezetimibe	%

Indicate, in each clinical scenario proposed, in what percentage you use each of the pharmacological strategies mentioned:

	Cardiovascular disease (%)	Diabetes Mellitus (%)	Dyslipidemia without CVD or DM (%)

Lifestyle modification, only			
High-intensity statin* and ezetimibe	%		
Moderate-intensity statin^ and ezetimibe	%		
High-intensity statin*	%		
Moderate-intensity statin^	%		
Low-intensity statin#	%		
Fibrate	%		
Bempedoic			
Others	%		

* High-intensity statins*: atorvastatin 80mg and rosuvastatin 20mg*
^ Moderate-intensity statins*: fluvastatin prolib (80mg), pravastatin (40mg), lovastatin (40mg), simvastatin (10mg, 20mg and 40mg), pitavastatin (1mg, 2mg and 4mg), atorvastatin (10mg, 20mg and 40mg) and rosuvastatin (5mg and 10mg) simvastatin*
# Low-intensity statins*: Fluvastatin (10mg and 20mg), pravastatin (10mg and 20mg) and lovastatin (20mg)*

Therapeutic objectives

For the following scenarios, which do you consider an adequate LDL-C control objective?

Recurring events in the last 2 years	mg/dl
Very high risk	mg/dl
High risk	mg/dl
Moderate risk	mg/dl
Low risk	mg/dl

Indicate in what percentage you think that your patients achieve LDL objectives in the following scenarios:

Recurring events in the last 2 years	%
Very high risk	%
High risk	%
Moderate risk	%
Low risk	%

Diabetes Mellitus	%
Cardiovascular disease	%
Cardiovascular disease + Diabetes Mellitus	%
Chronic kidney disease	%

Adverse events

Indicate approximately how often your patients have presented adverse events associated with statins ___________%

In these cases, in what percentage have you had to modify the treatment with statins? ___________%

If you have modified the treatment, indicate in what percentage you have made use of the following strategies:

Discontinue the statin	%
Reduce the dose of the same statin	%
Switch to another statin	%
Switch to another drug	%
Indicate which you use most frequently:	
Switch to nutraceutical	%
Others:	%
Indicate which:	

According to your clinical practice, which statin do you consider the safest as regards adverse events? (please, choose only one)

- ☐ Rosuvastatin
- ☐ Atorvastatin
- ☐ Pitavastatin
- ☐ Simvastatin
- ☐ Lovastatin

- Pravastatin
- Fluvastatin
- They are all equal
